# Structural Insight into the Two-Step Mechanism of PAI-1 Inhibition by Small Molecule TM5484

**DOI:** 10.3390/ijms22031482

**Published:** 2021-02-02

**Authors:** Machteld Sillen, Toshio Miyata, Douglas E. Vaughan, Sergei V. Strelkov, Paul J. Declerck

**Affiliations:** 1 Laboratory for Therapeutic and Diagnostic Antibodies, Department of Pharmaceutical and Pharmacological Sciences, KU Leuven, B-3000 Leuven, Belgium; machteld.sillen@kuleuven.be; 2 Department of Molecular Medicine and Therapy, United Centers for Advanced Research and Translational Medicine, Tohoku University Graduate School of Medicine, Sendai 980-8577, Japan; miyata@med.tohoku.ac.jp; 3 Department of Medicine, Feinberg Cardiovascular Research Institute, Northwestern University Feinberg School of Medicine, Chicago, IL 60611, USA; d-vaughan@northwestern.edu; 4 Laboratory for Biocrystallography, Department of Pharmaceutical and Pharmacological Sciences, KU Leuven, B-3000 Leuven, Belgium; Sergei.Strelkov@kuleuven.be

**Keywords:** plasminogen activator inhibitor 1, cardiovascular disease, fibrinolysis, thrombolysis, PAI-1 inhibitor, X-ray crystallography

## Abstract

Plasminogen activator inhibitor-1 (PAI-1), a key regulator of the fibrinolytic system, is the main physiological inhibitor of plasminogen activators. By interacting with matrix components, including vitronectin (Vn), PAI-1 plays a regulatory role in tissue remodeling, cell migration, and intracellular signaling. Emerging evidence points to a role for PAI-1 in various pathological conditions, including cardiovascular diseases, cancer, and fibrosis. Targeting PAI-1 is therefore a promising therapeutic strategy in PAI-1-related pathologies. A class of small molecule inhibitors including TM5441 and TM5484, designed to bind the cleft in the central β-sheet A of PAI-1, showed to be potent PAI-1 inhibitors in vivo. However, their binding site has not yet been confirmed. Here, we report two X-ray crystallographic structures of PAI-1 in complex with TM5484. The structures revealed a binding site at the flexible joint region, which is distinct from the presumed binding site. Based on the structural analysis and biochemical data we propose a mechanism for the observed dose-dependent two-step mechanism of PAI-1 inhibition. By binding to the flexible joint region in PAI-1, TM5484 might restrict the structural flexibility of this region, thereby inducing a substrate form of PAI-1 followed by a conversion to an inert form.

## 1. Introduction

**Figure 1:**
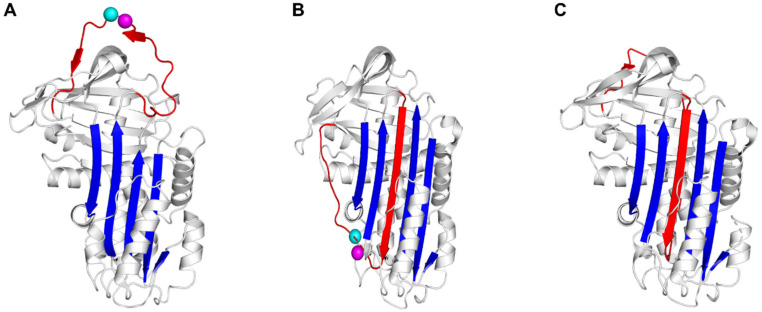
Cartoon representations of the structures of plasminogen activator inhibitor-1 (PAI-1) in the active (**A**), latent (**B**), or cleaved (**C**) conformation. PAI-1 is shown in white, the central PAI-1 β-sheet A in blue; the flexible reactive center loop (RCL) in red, and Arg346 and Met347 (P1–P1′) of the reactive center are indicated by magenta and cyan spheres, respectively. In latent (**B**) and cleaved (**C**) PAI-1, the N-terminal part of the RCL is inserted into the central PAI-1 β-sheet A to become strand 4 (s4A, shown in red). Protein Data Bank entries 1DB2 [11], 1DVN [12], and 3EOX [13] were used to generate this figure.

Plasminogen activator inhibitor-1 (PAI-1), a member of the serine protease inhibitor (serpin) superfamily, acts by inhibiting tissue-type (tPA) and urokinase-type (uPA) plasminogen activators (PAs). To this end, PAI-1 exposes a flexible reactive center loop (RCL) at its surface, which contains the substrate-mimicking peptide sequence Arg346-Met347 (designated as P1–P1′) (Figure 1A). Upon initial interaction, a non-covalent Michaelis complex is established through several interactions involving exosites and the RCL in PAI-1 [1,2]. Once the RCL is bound in the active site cleft of the PA, the P1–P1′ bond is attacked by the active site serine to generate a covalent acyl-enzyme intermediate [3]. Simultaneously, the N-terminal part of the cleaved RCL is inserted into the central β-sheet A of PAI-1 [4]. This major conformational change coincides with the translocation of the bound PA to the opposite side of the PAI-1 molecule. At the final stage of the inhibitory reaction, the PA is trapped into an irreversible complex by distortion of its catalytic triad [5,6]. Since serpin/protease interactions follow a branched pathway mechanism, the PAI-1/PA interaction can also result in the release of regenerated PA from cleaved PAI-1 through hydrolysis of the acyl-enzyme intermediate (Figure 1C) [7]. In contrast to other serpins, PAI-1 has the unique ability to spontaneously convert into a stable latent form by inserting the RCL segment N-terminal to the P1–P1′ cleavage site (residues 331 to 346, designated as P16-P1) in the core β-sheet A without prior cleavage to become strand 4 (s4A) (Figure 1B). This transition occurs with a half-life of approximately two hours at 37 °C in vitro. Importantly, high-affinity binding of cofactor vitronectin (Vn) to the flexible joint region in PAI-1, an area defined by helix E (hE), helix F (hF), and strand 1 of β-sheet A (s1A), slows down this latency transition in vivo [8,9,10].

Apart from its function in several biological processes, including fibrinolysis and wound healing, several studies revealed pleiotropic roles in various pathophysiological processes, e.g., cardiovascular disease, fibrosis, and cancer, as well as in age-related diseases [14,15,16,17]. Furthermore, experimental animal studies in which PAI-1 activity was inhibited provided evidence for beneficial effects of PAI-1 inhibition in vivo [18,19,20,21]. In the prospect of treating PAI-1-related pathologies, several groups have invested in the development of PAI-1 inhibitors, which include small organochemical molecules, peptides, monoclonal antibodies, and antibody fragments. Most of the small molecule inhibitors have been discovered by high-throughput screening of large libraries composed of small chemicals and natural products [22,23]. However, a major drawback of high-throughput screening for PAI-1 inhibitors is the frequently seen lack of efficacy when translated into in vivo settings, mainly due to poor activity against Vn-bound PAI-1 or selectivity issues. To address this limitation, Izuhara et al. [20] virtually screened a library of commercially available chemicals by applying two filters representing (I) the general drug-likeliness based on clinically used drug molecules, and (II) the specific lead-likeliness based on known PAI-1 inhibitors, including the peptide corresponding to P14-P10 (333-TVASS-337) of the RCL and inhibitors that bind close to the Vn binding site in PAI-1. Next, the selected compounds were computationally docked on PAI-1, focusing on the cleft in β-sheet A that is occupied by the RCL following loop insertion. Ninety-five candidates were able to fit the PAI-1 cleft. TM5007, the most effective compound, showed to be specific for the PAI-1/PA system and was furthermore effective in in vivo models of thrombosis and fibrosis [20]. Structure-activity relationship studies on TM5007 identified TM5275 as a derivative with an improved inhibitory profile and better oral bioavailability [24]. A similar docking simulation suggested that TM5275 binds within the cleft between the strands of β-sheet A, albeit at a different position compared to its precursor TM5007. Whereas TM5007 docked into the space occupied by P8-P3 (corresponding to 338-TAVIVS-344 of the RCL) of s4A in the latent form, TM5275 docked at the P14-P9 position of s4A. The differences in their presumed binding sites within the cleft also seemed to correlate with their mechanisms of action, i.e., by either preventing PAI-1/PA complex formation (TM5007) or inducing substrate behavior of PAI-1 (TM5275) [20,24]. Further structure optimization by substituting the lipophilic moiety and varying the acyl-type linker length led to the discovery of smaller derivatives, including TM5441 and TM5484 [25]. The beneficial effects of both compounds in several in vivo disease models, including models of stroke [26], cancer [27], multiple sclerosis [19], obesity [28,29], and other age-related pathologies [18,30,31], demonstrate their therapeutic potential.

Although these compounds have originally been designed to bind the central β-sheet A cleft, there is no experimental evidence that confirms their binding site or their mechanism of action. To understand their binding mechanism and how this interaction results in a modulation of PAI-1 activity, we initiated X-ray crystallographic studies by employing both soaking and co-crystallization strategies. Through soaking, we were able to solve several crystal structures of PAI-1 in complex with TM5484. The structures reveal a binding site at the flexible joint region in PAI-1.

## 2. Results and Discussion

### 2.1. Mechanism of Compound-Induced PAI-1 Inhibition

**Figure 2:**
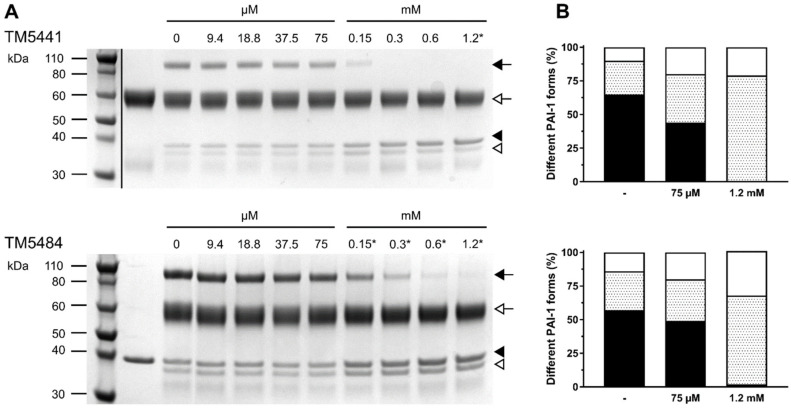
The effects of TM5441 and TM5484 on the outcome of the PAI-1/PA reaction. (**A**) SDS-PAGE analysis of the products of the PAI-1/tissue-type plasminogen activator (tPA) reaction in the presence of increasing concentrations of TM5441 or TM5484. Positions of SDS-stable PAI-1/PA inhibitory complex (black arrow), tPA (white arrow), unreactive/latent PAI-1 (black arrowhead), and cleaved PAI-1 (white arrowhead) are indicated to the right of the gels. The ladder in the top image was rearranged to the left side of the image. The lanes on the right side of the ladder correspond to tPA alone (upper panel) or PAI-1 alone (lower panel). For the compound concentrations indicated by an asterisk, minor precipitation was observed during the dilution of the compound; (**B**) conformational distribution of PAI-1 upon compound-induced inhibition in the absence (-) or presence of 75 µM and 1.2 mM of TM5441 (upper graph) or TM5484 (lower graph). Black bars represent SDS-stable PAI-1/tPA complex, dotted bars represent unreactive or latent PAI-1, and white bars represent cleaved PAI-1.

To characterize the inhibition of PAI-1 by TM5441 and TM5484, sodium dodecyl sulfate-polyacrylamide gel electrophoresis (SDS-PAGE) analysis of the reaction products of the PAI-1/tPA reaction was performed in the presence of the respective compounds. In the absence of either compound, the majority of PAI-1 formed a covalent inhibitory complex with tPA (~110 kDa), which could be distinguished from the smaller fractions of unreactive or latent PAI-1 (~43 kDa) and cleaved PAI-1 that lacks the C-terminal 33 residues (~40 kDa) (Figure 2A). In the presence of increasing concentrations of TM5441, the loss of a covalent PAI-1/tPA complex coincided with a large increase of unreactive PAI-1, and to a lesser extent of cleaved PAI-1 (Figure 2A,B). A similar effect was observed in the presence of TM5484 (Figure 2A), even though there seems to be a slightly larger increase in cleaved PAI-1 as compared to TM5441 (Figure 2B). Presuming that both compounds bind into the central cleft in β-sheet A, as was suggested for their precursor TM5275 by previous docking simulations [20,21,24], this could indicate a slightly different position for each compound inside the cleft. Indeed, it was previously shown that insertion of a peptide mimicking the C-terminal part of the RCL (P8-P3) at the bottom of β-sheet A hinders PAI-1/PA complex formation and might accelerate the conversion to the latent form of PAI-1 [32]. In contrast, a peptide corresponding to P14-P9, which binds at the top of β-sheet A, induces substrate behavior of PAI-1. A similar mechanism was observed for TM5275, which docks at the P14-P9 position and redirects the PAI-1/PA reaction towards the substrate branch. Even though TM5441 and TM5484 are both derived from TM5275, they mainly operate by preventing PAI-1/PA complex formation or by promoting the active-to-latent transition. The slightly larger fraction of cleaved PAI-1 observed with TM5484 might indicate that it binds in between the positions for TM5441 and TM5275 inside the central cleft.

Of note, at lower compound concentrations, the predominant mechanism of PAI-1 inactivation is to induce hydrolysis of the PAI-1/PA intermediate, which results in the release of PA from cleaved PAI-1. However, at high compound concentrations, TM5441 and TM5484 block PAI-1 activity by an alternate mechanism, i.e., by converting PAI-1 to an unreactive form. A similar mechanism was described for other PAI-1 neutralizing molecules, including the extensively studied tiplaxtinin or PAI-039 [33]. Site-directed mutagenesis and molecular modeling restricted the binding site of tiplaxtinin to a cleft in the flexible joint region defined by helices D and E (hD and hE) and strand 1 of β-sheet A (s1A), located immediately adjacent to the Vn binding site on PAI-1. Interestingly, within the same docking simulation that was undertaken for TM5007 and TM5275, tiplaxtinin bound at almost the same site as TM5007 in the central β-sheet A cleft. Therefore, binding of TM5441 and TM5484 might not be restricted to the central cleft as well.

### 2.2. Crystallographic Analysis of Compound-Bound PAI-1 Complexes

**Figure 3:**
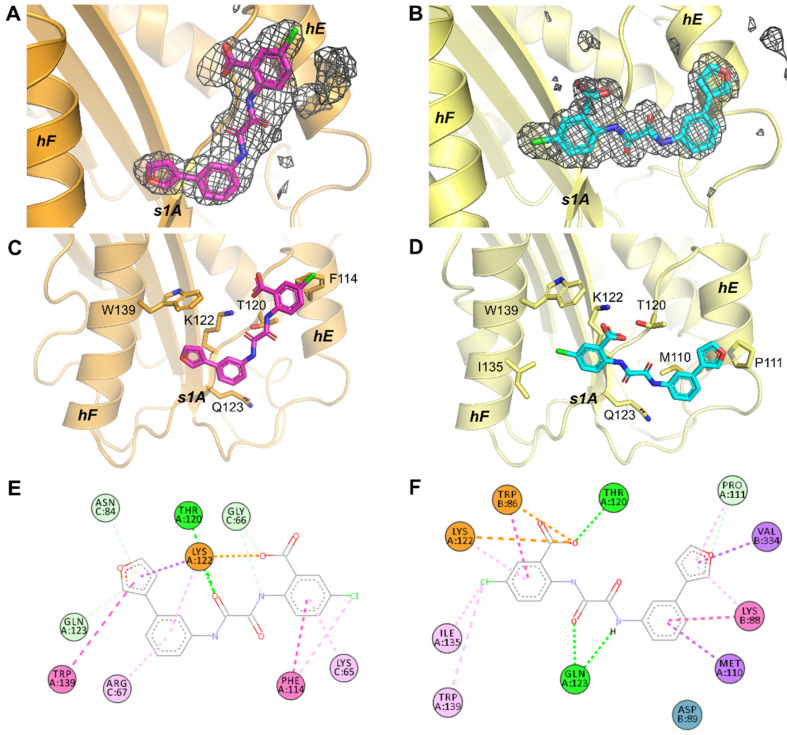
X-ray crystallographic structures of TM5484 bound to PAI-1. (**A**) Phenix.polder difference maps contoured at 4 sigma for TM5484 inside the PAI-1-W175F/Nb42/Nb64 crystal. PAI-1-W175F is colored orange and TM5484 is colored magenta. (**B**) Phenix.polder difference maps contoured at 4 sigma for TM5484 inside the PAI-1-stab crystal. PAI-1-stab is colored yellow and TM5484 is colored cyan. (**C**,**D**) Cartoon representation of the structure of the TM5484-bound PAI-1-W175F/Nb42/Nb64 complex and TM5484-bound PAI-1-stab. PAI-1-W175F is shown in orange and PAI-1-stab in yellow. TM5484 associated with PAI-1-W175F is colored magenta, whereas TM5484 associated with PAI-1-stab is colored cyan. Side-chains of the residues engaged in interactions with TM5484 are indicated and shown as sticks. (**E**,**F**) Two-dimensional diagrams of the relevant non-covalent protein-compound interactions as detected and visualized by Discovery Studio Visualizer. (**E**) Interactions between TM5484 and PAI-1-W175F. Residues in PAI-1-W175F are indicated by prefix “A” and residues in Nb64 of the neighboring asymmetric unit (ASU) are indicated by prefix ‘C’; (**F**) Interactions between TM5484 and PAI-1-stab. Residues in one PAI-1-stab molecule are indicated by prefix “A” and residues in the PAI-1-stab molecule of the neighboring ASU are indicated by prefix “B”. Conventional hydrogen bonds are presented as green dashed lines. Carbon hydrogen bonds and π-donor hydrogen bonds are presented as light green dashed lines. Salt bridges (Lys122) and pi-anion interactions (Trp86) are presented as orange dashed lines. ππ and amide-π stacking interactions are presented as pink dashed lines. π-alkyl and alkyl interactions, including edge-on Cl-π interactions, are presented as light pink dashed lines. π-sigma interactions are presented as purple dashed lines. Asp89 (blue) is involved in a van der Waals interaction.

To obtain further insight into the inhibitory mechanism of TM5441 and TM5484, X-ray crystallographic studies of the compounds in complex with PAI-1 were initiated. After crystallization screening of two stabilized active PAI-1 mutants, PAI-1-W175F and PAI-1-N150H-K154T-Q301P-Q319L-M354I (PAI-1-stab), free or in complex with PAI-1 neutralizing nanobodies (Nb42 and Nb64), diffracting crystals were obtained and soaked with the compounds. Soaked crystals of the PAI-1-W175F/Nb42/Nb64 complex and unbound PAI-1-stab readily diffracted to 2.3 Å or better (Table 1). Even though soaking was performed for both compounds, the resulting calculated electron density maps enabled unambiguous positioning of TM5484 only, independent of the soaking time. Compared to TM5484, TM5441 contains a slightly longer linker and possibly, due to its bigger size, could not enter the same confined binding area, as was observed for TM5484, due to steric restrictions of neighboring molecules inside the three-dimensional crystal. Two nearly identical crystal structures (root-mean-squared deviations for all Cα atoms (Cα RMSD) of 0.8 Å) of the TM5484-bound PAI-1-W175F/Nb42/Nb64 complex were obtained after two hours (Figure 3A) as well as overnight soaking (not reported), and one structure of TM5484-bound PAI-1-stab was obtained after overnight soaking (Figure 3B). For crystals of the PAI-1-W175F/Nb42/Nb64 complex, one ternary complex was present in the asymmetric unit (ASU). In the case of the PAI-1-stab crystal, two PAI-1 molecules (chains A and B) were observed of which only one had TM5484 compound bound (chain A). Indeed, in the case of the second PAI-1 molecule (chain B), access to the binding site is restricted due to close crystallographic contacts with a PAI-1 molecule from the neighboring ASU. Alignment of the two PAI-1 molecules showed that they have nearly identical conformations (Cα RMSD of 0.79 Å, excluding the flexible RCL).

**Figure 4:**
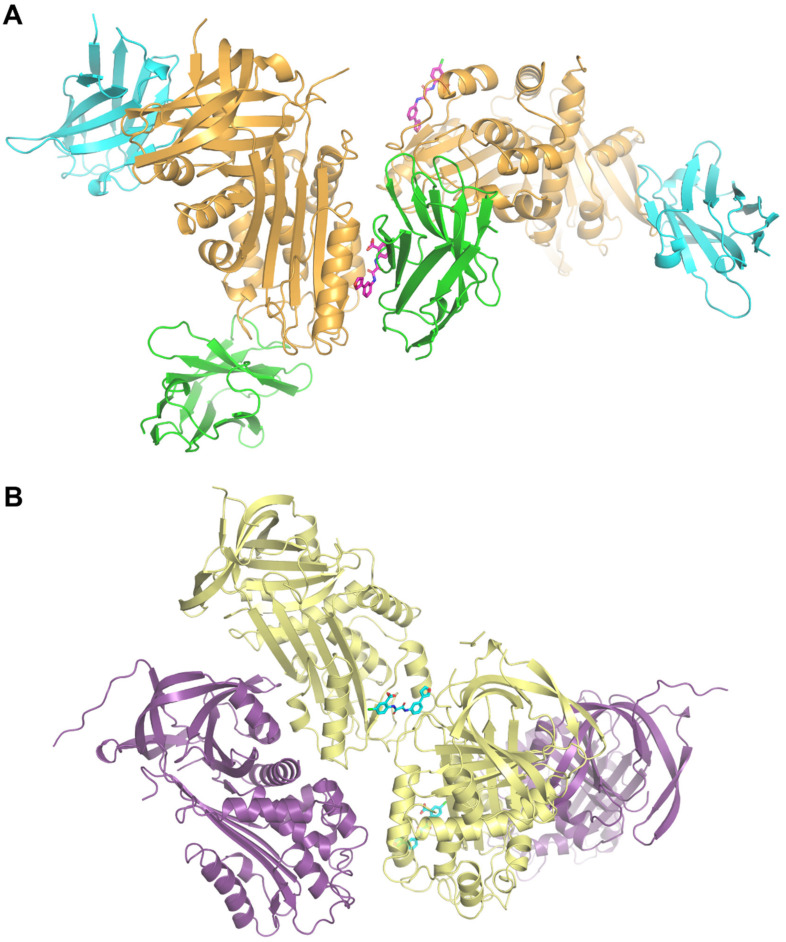
Cartoon representation of the PAI-1/TM5484 complexes. (**A**) In the case of the two PAI-1-W175F/Nb42/Nb64 crystals, TM5484 is located in the same orientation at the crystallographic interface between one PAI-1 molecule and an Nb64 molecule of the neighboring ASU. PAI-1 is shown in orange, Nb42 in cyan, Nb64 in green and TM5484 in magenta. (**B**) The ASU in the PAI-1-stab crystal comprises two PAI-1-stab molecules and one TM5484 compound associated with one of the two PAI-1 molecules. TM5484 is located at the crystallographic interface between one PAI-1 molecule of ASU 1 and one PAI-1 molecule of a neighboring ASU. PAI-1 molecules inside one ASU are shown in yellow and purple. TM5484 is shown in cyan.

Comparison of TM5484 in the compound-bound structures shows TM5484 at the crystallographic interface between PAI-1 and Nb64 (in the PAI-1/Nb42/Nb64 crystal, Figure 4A), or between two PAI-1 molecules (in the PAI-1-stab crystal, Figure 4B). Importantly, in either case the TM5484 molecule is located at the flexible joint region in PAI-1, an area that is defined by α-helices hE, hF, and s1A (Figure 3C,D).

Comparison of the TM5485-bound PAI-1/Nb42/Nb64 and PAI-1-stab structures revealed that the TM5484 molecule bound in two different orientations (Figure 3C,D), hereafter referred to as “orientation 1” (Figure 3C) and “orientation 2” (Figure 3D). The different binding modes observed in the different crystal systems are most likely caused by steric restrictions due to crystal packing. However, the functional groups of the compound that were previously identified as essential for the interaction with PAI-1 remain importantly involved. Studies undertaken to investigate the structure-activity relationship of the precursors of TM5484 suggested that the carboxylic acid group was essential to bind PAI-1, whereas the bulky lipophilic group has a secondary effect [24,25]. In this respect, it is notable that the carboxylic acid interacts with PAI-1 Lys122 (s1A) through the formation of a salt bridge independent of the orientation of TM5484 (Figure 3E,F), and with PAI-1 Thr120 (s1A) through an additional hydrogen bond in orientation 2 (Figure 3F). In orientation 1, the Cl-atom substituted on the same phenyl group is involved in an edge-on Cl–π interaction with Phe114 in hE of PAI-1 (Figure 3E). With the nearest aromatic atom at 3.5 Å and a distance of 4.7 Å to the Phe114 ring centroid, the interaction approaches the average distances (3.6 and 4.3 Å, respectively) that were reported for edge-on Cl–Phe interactions [34]. In orientation 2, the Cl-atom is located 4.3 Å away from the sidechain of Trp139 in hF and 5.4 Å away from the ring centroid in an edge-on geometry, thus resulting in weaker interactions. Additionally, the Cl-atom makes a 3.4 Å van der Waals interaction with the side chain of Ile135 in hF (Figure 3F). Through the furan group, TM5484 forms a non-classical carbon hydrogen bond (weaker H-bond) with the side-chain of PAI-1 Gln123 (s1A) in orientation 1 (Figure 3E) or with Pro111 (hE) in orientation 2 (Figure 3F). Furthermore, the phenylfuran group engages in hydrophobic interactions (π-sigma, π-alkyl, and π–π stacking interactions) with Lys122 (s1A) and Trp139 (hF) in orientation 1 (Figure 3E), or with PAI-1 Met110 and Pro111 (hE) in orientation 2 (Figure 3F). In both orientations, binding of TM5484 to s1A is further stabilized by hydrogen bonds involving the linker (Figure 3E,F).

As mentioned, TM5484 is located at the crystallographic interface. Apart from direct interactions with the flexible joint region of PAI-1, TM5484 also makes contacts with molecules of the neighboring ASUs (Figure 4). In orientation 1, these interactions are limited to weaker hydrogen bonds with Nb64 residues Gly66 and Asn84 and hydrophobic interactions involving the side chains of Lys65 and Arg67 in Nb64 (Figure 3E). In orientation 2, however, the neighboring PAI-1-stab molecule forms a π–π stacking interaction and an electrostatic π-anion interaction with the chloroanthranilic acid moiety of TM5484 through Trp86. Furthermore, Lys88 and Val334 engage in hydrophobic interactions (π-alkyl and amide-π stacking, and π-sigma, respectively) with the phenylfuran moiety of TM5484 (Figure 3F).

**Figure 5:**
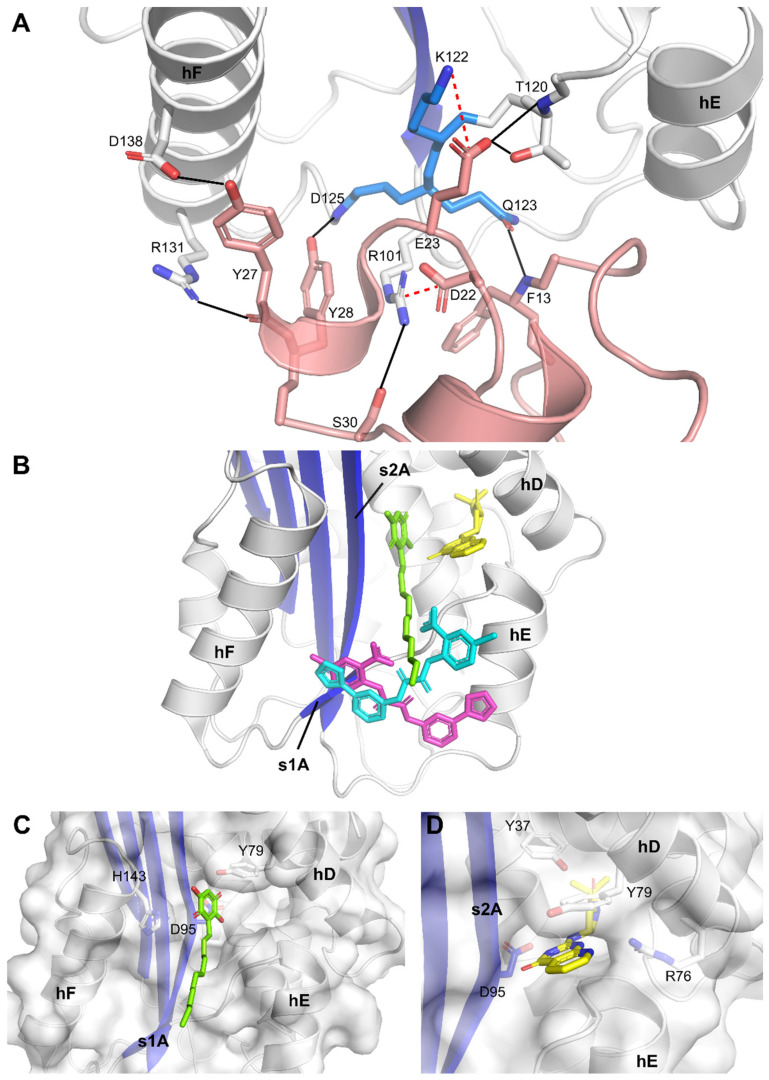
X-ray crystallographic structures of PAI-1 bound to vitronectin (Vn) and small molecule PAI-1 inhibitors. (**A**) Detail of the interaction between active PAI-1 and the somatomedin B (SMB) domain of Vn (pink, Protein Data Bank (PDB) ID: 1OC0 [8]). Hydrogen bonds are presented as black lines and salt bridges as dashed lines; (**B**) superimposition of the published crystal structures of PAI-1 in complex with embelin (green) and AZ3976 (yellow) with the structures of TM5484 bound to PAI-1/Nb42/Nb64 (magenta) and to PAI-1-stab (cyan); (**C**) cartoon representation of embelin (green) bound to a groove aligned by hD, hF, s2A and the hE-s1A loop in active PAI-1 (PDB ID: 3UT3 [38]); (**D**) cartoon representation of AZ3976 (yellow) bound to a deep pocket aligned by hD and s2A in latent PAI-1 (PDB ID: 4AQH [39]). PAI-1 is colored white with the central β-sheet A shown in blue.

Importantly, the binding region observed in the crystal structures was formerly identified as the primary high-affinity binding site for Vn [8]. Through a hydrogen bond and salt bridge network, the somatomedin B (SMB) domain of Vn interacts with PAI-1 residues Arg101 (hE-s2A loop); Thr120, Lys122, Gln123, and Asp125 (s1A); and Asp138 (hF) (Figure 5A). Furthermore, PAI-1 residues Pro111 and Phe114 (hE); Val124 (s1A); and Ile135 and Trp139 (hF) are directly engaged in the largely hydrophobic interface. Since our structures reveal that TM5484 interacts with several of these residues, TM5484 may possibly prevent binding of Vn or expel Vn from the complex with PAI-1. Furthermore, the mechanism observed using SDS-PAGE analysis, i.e., inducing PAI-1 substrate behavior and impairing its reaction with PAs may result from a decreased flexibility in the flexible joint region and from allosteric modulation of PAI-1. Indeed, by interacting with residues in hE, s1A, and hF, TM5484 links these structural elements and may hamper (I) the outward movement of hF that is required for full insertion of the RCL during the inhibitory reaction between PAI-1 and PAs and (II) the sliding movement of the s2A and s3A into the flexible joint region, which is required to expand the central β-sheet A and accommodate the RCL as s4A. This mechanism was previously described for Vn and other small molecule inhibitors that bind in the same region, including tiplaxtinin [33,35]. Even though experiments involving Vn showed that tiplaxtinin lost its PAI-1 neutralizing activity when PAI-1 makes a complex with Vn, tiplaxtinin exhibited in vivo activity in several animal models [36,37]. Thus, the possible binding of TM5484 to the Vn binding site in PAI-1 is not necessarily in conflict with its proven in vivo efficacy [19].

Furthermore, a region adjacent to the observed TM5484 binding site has been implicated in binding several other small molecules (Figure 5B). Crystallographic studies, molecular modeling, and mutagenesis studies identified a hydrophobic area in the flexible joint region as a common organochemical compound-binding pocket. Several negatively (AR-H029953XX, ANS, Bis-ANS) and positively (XR5118) charged amphipathic inhibitors have been shown to bind overlapping but non-identical sites within this hydrophobic groove, resulting in variable induced molecular changes in PAI-1 and in a differential susceptibility to Vn-bound PAI-1 [40]. Whereas both groups inhibit PAI-1 via a two-step mechanism involving a rapid reversible conversion into a PAI-1 form exhibiting substrate behavior and a slower irreversible conversion into an unreactive form, only the negatively charged inhibitors caused the unreactive PAI-1 to polymerize. Embelin, which has a polar aromatic ring structure, conjugated with an aliphatic chain, interferes with PAI-1 functionality by a mechanism similar to that for the negatively charged inhibitors [38]. Mutagenesis studies and co-crystallization of embelin with the stabilized active PAI-1-14-1B mutant (PAI-1-N150H-K154T-Q319L-M354I) pointed out key interactions with hD (Tyr79), s2A (Asp95), and hF (His143) residing in a small and charged groove adjacent to a larger and deeper hydrophobic pocket (Figure 5C). Interestingly, this deeper pocket is accessible in the (pre)latent form of PAI-1 and has been shown to accommodate AZ3976, an azetidine derivative that accelerates the active-to-latent transition of PAI-1 (Figure 5D) [39]. In contrast to AZ3976 that was crystallized with the latent form of PAI-1, PAI-1 inside the crystal structure of embelin-bound PAI-1 and our structures of TM5484-bound PAI-1 represents the active conformation. Furthermore, comparison of the crystal structure of embelin-bound PAI-1 and TM5484-bound PAI-1 reveals no major structural differences (Cα RMSD of 0.6 Å) or any change in the PAI-1 molecule that could explain the inert behavior of PAI-1. It should be noted that our crystal structure results from soaking experiments with preformed well-ordered crystals of PAI-1. Polymerization of PAI-1 or the conversion to latent PAI-1 upon binding of the compound requires large conformational changes and would disrupt the crystals. Therefore, these inert forms could not be observed in our crystal structures.

In conclusion, viewing our mechanistic and crystallographic data in the context of the reported activity of other small organochemicals indicates that TM5484 inhibits PAI-1 activity in a dose-dependent two-step manner, i.e., by inducing a substrate form of PAI-1 followed by a conversion to an inert form. Structural analysis of PAI-1 crystals soaked with a solution containing TM5484 allowed us to unambiguously identify its binding site similar to the Vn-binding region comprising hE, hF, and s1A. Binding of TM5484 to this region in PAI-1 might restrict the structural flexibility of the flexible joint region that is normally required for PAI-1 functionality, hence providing a rationale for its inhibitory mechanism. In addition, the observed mechanism also suggests that TM5484 may shorten the functional half-life of PAI-1 and may affect its clearance rate, which likely has an impact on the perceived in vivo efficacy of this small molecule inhibitor.

## 3. Materials and Methods

### 3.1. Cloning, Expression, and Purification of PAI-1 and Nbs

The expression and purification of PAI-1-W175F, PAI-1-stab, Nb42, and Nb64 were performed as previously described **[41,42]**. Briefly, the proteins were produced in the *Escherichia coli* Rosetta^TM^ 2(DE3)pLysS strain (Merck) as His_6_-tagged SUMO fusion proteins in auto-induction ZYP-5052 media [43]. The cell pellet was resuspended in buffer (50 mM sodium phosphate, 250 mM NaCl, 12.5 mM imidazole, pH 7 (PAI-1) or pH 7.5 (Nbs)). The fusion proteins were purified from the cell lysate using a 5 mL HisTrap HP column (GE Healthcare, Chicago, IL, USA) and treated with SUMO-hydrolase (1:250 mass ratio protein:SUMO hydrolase) to remove the His_6_-tagged SUMO domain. Subsequently, this mixture was loaded on a 5 mL HisTrap HP column and the flow through containing the respective non-tagged PAI-1 variant or Nb was collected, dialyzed to ion exchange buffer (20 mM sodium phosphate, pH 7.5), loaded onto a 10 mL HiTrap SP column (GE Healthcare), and eluted by a 0–1 M NaCl gradient. Subsequently, fractions containing PAI-1 or Nb were collected and concentrated using a Vivaspin 15R centrifugal concentrator with a 10 kDa molecular mass cut-off (Sartorius) and further purified by gel filtration on a HiLoad 26/60 Superdex 200 column (GE Healthcare) equilibrated in 20 mM Bis-Tris, 300 mM NaCl, pH 6. All purification steps were conducted on ice or at 4 °C.

### 3.2. Determination of the Conformational Distribution of PAI-1 upon Compound-Induced Inhibition

The effects of TM5441 and TM5484 on the conformational distribution of PAI-1 during its reaction with tPA were analyzed by SDS-PAGE. First, a serial two-fold dilution range in phosphate-buffered saline at pH 7.4 was prepared from the 120 mM stock solutions of the compounds in 100% DMSO. After two subsequent dilution steps, this resulted in a final concentration ranging from 1.2 mM to 9.4 µM for each compound, containing 1% DMSO. Next, 5 µL of the compound was incubated with an equal volume of 4.6 µM wild type PAI-1 for 10 min at 37 °C. Next, 10 µL of 6.6 µM tPA was added and the sample was incubated for 30 min at 37 °C. Samples were analyzed by SDS-PAGE using NuPAGE Novex 4–12% Bis-Tris protein gels (Invitrogen) and stained by Coomassie brilliant blue. The amount of PAI-1 (unreactive plus latent, cleaved, and complexed with PA) was estimated from the intensity of the corresponding bands by densitometric scanning using the Bio-Rad ChemiDoc MP imager and Image Lab software (Bio-Rad, Hercules, CA, USA).

### 3.3. Crystallization and Data Collection

Crystals of the PAI-1-W175F/Nb42/Nb64 complex were prepared as previously described [41]. Suitable crystals were transferred to a soaking solution containing 0.1 M Bis-Tris pH 6.5, 10% *w/v* PEG 3350, 20% *v/v* ethylene glycol, 0.8–3% *v/v* DMSO, and 2 mM compound, and incubated for either two hours or overnight before harvesting in a cryo-loop and flash freezing in liquid nitrogen. Prior to the crystallization of PAI-1-stab, PAI-1-stab was buffer exchanged into 20 mM Bis-Tris pH 6 containing 300 mM sodium chloride and concentrated to 10 mg/mL. Crystals of PAI-1-stab were grown using the hanging-drop vapor diffusion technique at 20 °C by mixing 2 µL of protein solution and 1 µL of precipitant consisting of 0.2 M trisodium citrate and 10% *w/v* PEG 3350. Before mounting and flash freezing in liquid nitrogen, the crystals were transferred to a soaking solution containing 0.2 M trisodium citrate, 10% *w/v* PEG 3350, 20% *v/v* ethylene glycol, 0.8% *v/v* DMSO, and 2 mM compound.

In parallel, co-crystallization experiments were conducted by incubating PAI-1-stab with a 1.2-fold molar excess of the compound. The PAI-1-stab/TM5441 complex was crystallized at 9.5 mg/mL using the hanging-drop vapor diffusion technique at 20 °C by mixing 1 µL of protein solution with 1 µL of crystallization solution containing 0.1 M Bis-Tris pH 6.5 and 2.4 M di-ammonium sulfate. The PAI-1-stab/TM5484 complex was crystallized at 8.4 mg/mL, using a crystallization solution consisting of 0.1 M Bis-Tris pH 6.5, 0.1 M sodium chloride and 1 M di-ammonium sulfate. Before flash freezing in liquid nitrogen, crystals of the aforementioned complexes were cryo-protected by briefly transferring to their respective crystallization solutions supplemented with 20% *v/v* ethylene glycol.

X-ray diffraction data collection was performed at 100 K using the PROXIMA1 beamline at synchrotron Soleil (Paris, France). Data collection and refinement statistics are listed in Table 1.

### 3.4. Structure Determination, Refinement, and Analysis

The obtained diffraction data were processed using autoPROC in default settings, with a high-resolution cutoff on *CC*_1/2_ 0.60 [44]. The data were initially phased by molecular replacement using the previously published structures of the PAI-1-W175F/Nb42/Nb64 complex (Protein Data Bank (PDB) ID 6GWN [41]) or the active form of PAI-1-stab (PDB ID 6GWP, chain A [41]) as search models. Next, the compound was modeled using the ligand builder Lidia in Coot. A restraint file for the ligand was generated from the SMILES string using eLBOW [45] available in phenix. The structures of the compound-bound complexes were improved by iterative rounds of manual model building using Coot [46] and refined using phenix.refine [47]. The final models of TM5484-bound PAI-1-W175F/Nb42/Nb64 and PAI-1-stab have been deposited to the PDB under the accession codes 7AQG and 7AQH, respectively. PyMOL (The PyMOL Molecular Graphics System, version 2.0.7, Schrödinger, LLC, New York, NY, USA) was used to visualize and superimpose models and to compute root-mean-squared deviations for all Cα atoms (Cα RMSD). Relevant non-covalent PAI-1−compound interactions were detected and visualized on a two-dimensional diagram using BIOVIA Discovery Studio Visualizer v20.1.0.19295. To support the positioning of the compound, polder difference maps [48] were generated using phenix.polder and contoured at 4 sigma.

## Figures and Tables

**Table 1:** Data collection and refinement statistics.

	PAI-1-W175F/Nb42/Nb64 + TM5484 (2 h Soaking)	PAI-1-Stab + TM5484
**Data collection**		
Space group	*P* 2	*C* 2
Cell parameters		
a, b, c (Å)	45.5, 71.5, 96.2	135.3, 64.3, 106.6
α, β, γ (°)	90, 101.3, 90	90, 117, 90
Resolution range (Å)	36.15–2.27 (2.35–2.27)	33.44–1.77 (1.83–1.77)
*R_merge_*	0.069 (0.603)	0.087 (1.07)
*I*/*σ (I)*	9.44 (2.02)	9.03 (1.99)
*CC*_1/2_	0.997 (0.83)	0.999 (0.812)
Completeness (%)	99.45 (99.60)	99.36 (98.78)
Redundancy	3.7 (3.7)	6.7 (7.0)
**Refinement**		
No. of PAI-1 molecules/ASU	1	2
Reflections used in refinement	27,958 (2759)	79,244 (7845)
Reflections used for *R_free_*	2083 (220)	3996 (407)
*R_work_*	0.214 (0.320)	0.178 (0.276)
*R_free_*	0.266 (0.361)	0.213 (0.322)
No. of non-hydrogen atoms	4637	6254
Protein	4561	5746
Ligands	27	27
Water	49	481
*B* factors (Å^2^)		
Protein	58.47	29.32
Ligands	56.09	33.82
Water	49.95	39.73
R.m.s. deviations		
Bond lengths (Å)	0.002	0.009
Bond angles (°)	0.51	0.95

Diffraction data were collected from a single crystal. The values in parentheses are for the highest resolution shell. ASU: asymmetric unit; R.m.s.: root-mean-squared.

## Data Availability

The final models of TM5484-bound PAI-1-W175F/Nb42/Nb64 and PAI-1-stab have been deposited to the Protein Data Bank under the accession codes 7AQG and 7AQH, respectively.

## Abbreviations

ASU	asymmetric unit
Cα RMSD	root-mean-squared deviations for all Cα atoms
h[X]	α-helix X
PA	plasminogen activator
PAI-1	plasminogen activator inhibitor-1
PAI-1-stab	PAI-1-N150H-K154T-Q301P-Q319L-M354I
PDB	Protein Data Bank
RCL	reactive center loop
s[x]A	strand number [x] of β-sheet A
SDS-PAGE	sodium dodecyl sulfate-polyacrylamide gel electrophoresis
tPA	tissue-type plasminogen activator
uPA	urokinase-type plasminogen activator
Vn	vitronectin
